# Current and Promising Antivirals Against Chikungunya Virus

**DOI:** 10.3389/fpubh.2020.618624

**Published:** 2020-12-15

**Authors:** Friederike I. L. Hucke, Joachim J. Bugert

**Affiliations:** Department of Virology, Bundeswehr Institute of Microbiology, Munich, Germany

**Keywords:** antiviral design, CHIKV therapy, direct antiviral action, host-targeting antiviral, comparison of *in vitro* efficacies, favipiravir, ribavirin

## Abstract

Chikungunya virus (CHIKV) is the causative agent of chikungunya fever (CHIKF) and is categorized as a(n) (re)emerging arbovirus. CHIKV has repeatedly been responsible for outbreaks that caused serious economic and public health problems in the affected countries. To date, no vaccine or specific antiviral therapies are available. This review gives a summary on current antivirals that have been investigated as potential therapeutics against CHIKF. The mode of action as well as possible compound targets (viral and host targets) are being addressed. This review hopes to provide critical information on the *in vitro* efficacies of various compounds and might help researchers in their considerations for future experiments.

## Introduction Chikungunya Virus

Chikungunya virus (CHIKV) is a single-stranded RNA virus with a positive sense genome of about 11,800 nucleotides. CHIKV structure and genome organization follow those of all alphaviruses. The virion has a lipid-bilayer envelope that is tightly associated with an icosahedral nucleocapsid shell (240 capsid copies) which encapsidates genomic RNA (1). The genome contains two open reading frames (ORFs), which encode the non-structural (ns) or replicase polyprotein and the structural polyprotein.

CHIKV is primarily transmitted to humans by the bite of an infected mosquito, mainly of the *Aedes* species. CHIKV causes the so-called chikungunya fever (CHIKF) which is characterized by high fever, headache and the hallmarks of the disease, myalgia and polyarthralgia (1). The latter especially can last for months or even years after the acute phase of the illness has passed, causing a severely deteriorated quality of life for the patient. The resulting stooped bearing and rigid gait of infected individuals are described in the word origin of the disease “kungunyala,” which is Makonde for “that which bends up.” CHIKV was first described in 1955 by Robinson and Lumsden after an outbreak in present-day Tanzania in 1952 (2).

**Figure 1:**
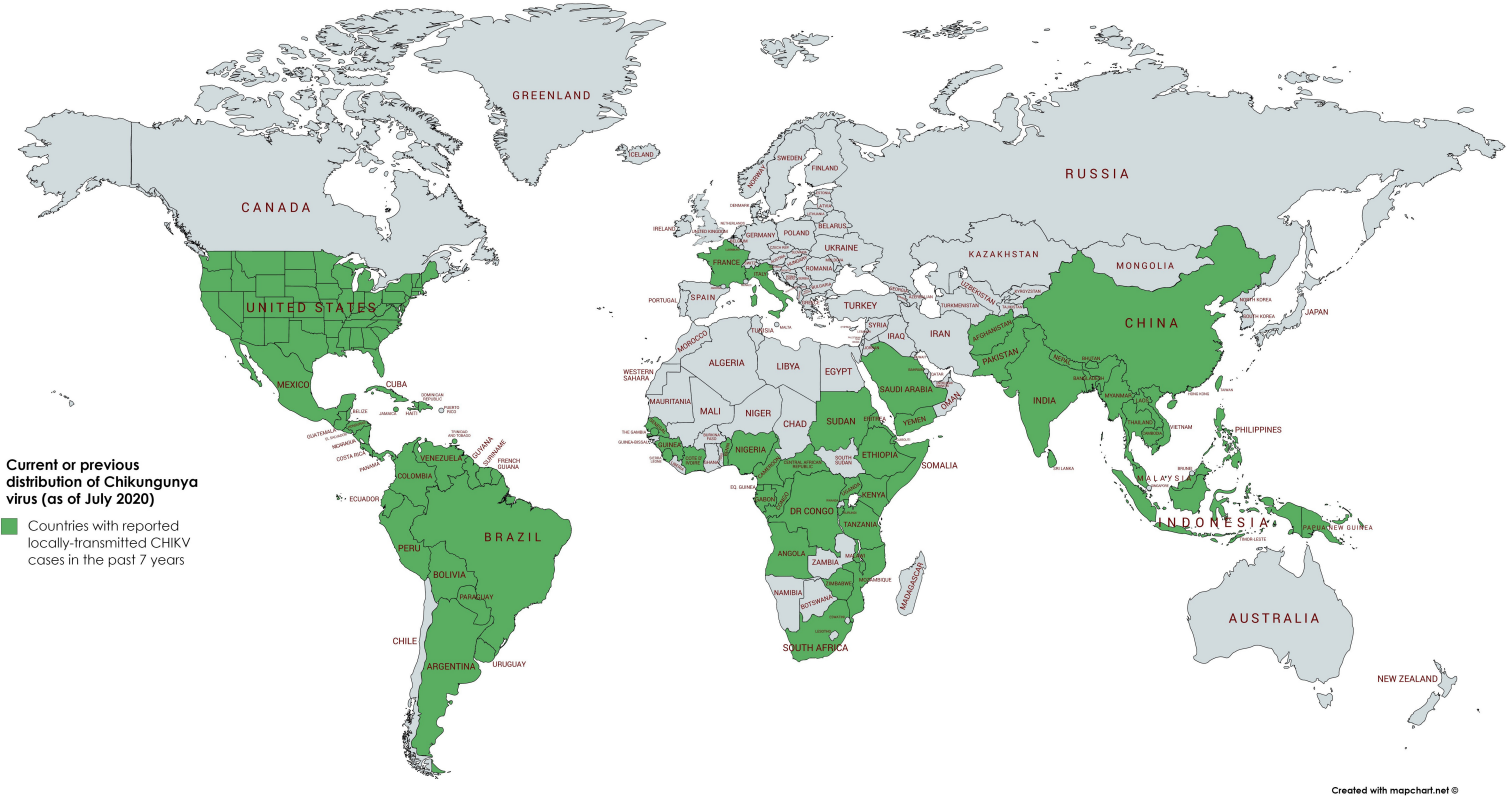
Geographical distribution of CHIKV. Word map showing the distribution of autochthonous CHIKV transmissions in the past 7 years (2013–2020) highlighted in green. In the continental United States of America, only travel associated cases have been reported in the past 3 years. As of July 2020, the latest autochthonous cases in Europe have been recorded in France and Italy in 2017 (3, 4).

Until 2004, CHIKV was mainly distributed in tropical and subtropical regions of sub-Sahara Africa and Southeast Asia. It caused sporadic outbreaks mainly during the rainy season. In 2004, however, a massive outbreak in Kenya led to close to half a million infected people. This epidemic initiated the spread to more than 22 countries, including countries with a moderate climate such as France and Italy (Figure 1) (5).

Following the bite of a CHIKV infected mosquito, the virus is transported to the nearest lymph node and transferred to monocytes and macrophages which enter the bloodstream. At this point, viremia sets in by the active infection of human blood monocytes and other peripheral blood mononuclear cells. CHIKV then reaches the muscles and joints, where the infection causes the main symptoms of CHIKF—myalgia and arthralgia (6). Apart from muscles and joints, CHIKV may also target a range of secondary organs and thus cause severe complications in patients (i.e., renal, respiratory, hepatic, cardiac, and neural syndromes) (7). As neither specific antiviral drugs nor a licensed vaccine are available, the therapy of CHIKF is based on supportive measures and the treatment of symptoms [non-steroid anti-inflammatory drugs (NSAIDs) and fluid therapy (8)].

For detailed information on CHIKV epidemiology, replication, disease mechanism, and prophylaxis, we refer to the reviews of Silva and Dermody (1), Pietila et al. (9), and Hucke et al. (10).

## Antivirals Against Chikungunya Virus

### Direct-Acting Antivirals

The following chapter will deal with various compounds that are or have been in the focus of research and showed some promising results *in vitro* mainly against CHIKV and/or other relevant alphaviruses. Various compounds made it to *in vivo* studies but so far there is no licensed therapeutic drug acting directly against CHIKV or any other alphavirus. There are many compounds which are currently under investigation for their anti-CHIKV efficacy. However, as the scope of this review is limited, we will only discuss compounds that either showed efficacy in a variety of *in vitro* assays or were repeatedly investigated by different (independent) research groups. It must be noted that compound efficacy can vary considerably, depending on the cell line, virus strain, or assay method that is being used. Table 1 illustrates this fact and gives an overview of EC_50_/CC_50_ values of common substances used as experimental controls in *in vitro* trials. For the interested reader I refer to the reviews of Abdelnabi et al. (38), Subudhi et al. (39) and the review of da Silva-Junior et al. (40), focussing on the medicinal chemistry of synthetic and natural compounds against CHIKV. Furthermore, the review of Bugert et al. (41) inspects antivirals against alphaviruses and other viral agents relevant in medical biodefence.

**Table 1:** Comparison of compounds with anti-CHIKV property.

**Compound**	**EC_**50**_ (μM)**	**CC_**50**_ (μM)**	**References**
**CHIKV entry inhibitors**			
Chloroquine (reference)	5–11	>36–100	(11–17)
Suramin	8.8–62.1	350 to >700	(18)
Suramin conjugates	1.9–2.7	50 to >200	(19)
**nsP1 inhibitors**			
Lobaric acid	5.3–16.3	50–76	(20)
[1,2,3]triazolo[4,5-d]pyrimidin-7(6H)-ones (lead)	<1.0	>668	(21)
[1,2,3]triazolo[4,5-d]pyrimidin-7(6H)-one (compound 8)	1.1–5.3	>300	(14)
**nsP2 inhibitors**			
Bassettos *in silico* lead (compound 1)	5	72	(12)
1,3-thiazolidin-4-one (compound 8)	1.5	>200	(22)
Compound ID1452-2	31	n.d.	(23)
**nsP4 inhibitors and inhibitors of viral genome replication**			
Ribavirin	2.05–756.8	49 to >500	(16, 24–29)
β-d-N4-hydroxycytidine (NHC)	0.2–1.8	2.5–30.6	(30)
Favipiravir (T-705)	16–245.13	>636	(25, 31)
Defluorinated Favipiravir (T-1105)	7–47	>571	(31)
Sofosbuvir	1–17	402	(24)
Mycophenolic acid (MPA)	0.5–1.6	370	(16, 24, 32)
**Protein kinase C inhibitors**			
Prostratin	0.2–8	50 to >100	(11, 33)
12-O-tetradecanoylphorbol 13-acetate (TPA)	0.0029	5.7	(11)
Phorbol-12,13-didecanoate	0.006	~4.1	(13)
12-O-decanoylphorbol 13-acetate (DPA)	2.4	4.6	(34)
12-O-decanoyl-7-hydroperoxy-5-ene-13-acetate phorbol	4.0	7.8	(34)
Neoguillauminin A	17.7	~35	(15)
12-deoxy phorbol Compound 1	0.13	12.7	(15)
12-deoxyphorbol Compound 2	0.02	4.85	(15)
12-deoxyphorbol Compound 4	0.02	30.0	(15)
Trigocherrin A	1.5	35	(17)
**Multiple/unidentified targets**			
Micafungin	17.2–20.63	>100	(35)
Abamectine	1.4 ± 0.9 (Huh-7.5) and 1.5 ± 0.6 (BHK-21)	15.2 ± 1.0 (Huh-7.5) and 28.2 ± 1.1 (BHK-21)	(36)
Ivermectine	1.9 ± 0.8 (Huh-7.5) and 0.6 ± 0.1 (BHK-21)	8.0 ± 0.2 (Huh-7.5) and 37.9 ± 7.6 (BHK-21)	(36)
Berberine	1.9 ± 0.9 (Huh-7.5) and 1.8 ± 0.5 (BHK-21)	>100 (Huh-7.5 and BHK-21)	(36)
coumarin derivatives conjugated with guanosine	9.9–13.9	96.5–212	(37)

*The above mentioned compounds have been in the focus of studies during the past 7 years (with the exception of the reference compounds chloroquine and ribavirin). The compounds have been arranged according to their (known) point of interaction. EC_50_ and CC_50_ may display a broad range due differences in cell line, virus strain, and assay method within the study. Unless stated otherwise, EC_50_ and CC_50_ were generated with Vero cell lines*.

*BHK, baby hamster kidney cells; CC50, cytotoxicity concentration 50%; CHIKV, Chikungunya virus; EC50, half maximal effective concentration; Huh, Human hepatocarcinoma cells; n.d., not determined; nsP, non-structural protein*.

#### CHIKV Entry Inhibitors

##### Chloroquine

Chloroquine is a licensed drug for the prophylaxis and treatment of malaria. Furthermore, it is prescribed for the treatment of systemic lupus erythematosus and rheumatoid arthritis (42). Chloroquine also shows *in vitro* antiviral activity against several viruses, such as human immunodeficiency virus (HIV), severe acute respiratory syndrome (SARS) coronavirus and alphaviruses (43). Khan demonstrated that chloroquine is able to inhibit CHIKV replication in VeroA cells in a dose-dependent manner. Apart from this mode of action, it is also assumed that the drug interferes with the endosome-mediated CHIKV internalization. Bernard et al. (44) showed that chloroquine raises the endosomal pH by interfering with the protonation of the endocytic vesicles and thereby prevents the E1 fusion step needed for the release of CHIKV RNA into the cell cytoplasm. Various research groups used chloroquine as a reference compound in their *in vitro* studies (Table 2) (11–15, 45).

**Table 2:** Efficacy of selected compounds against CHIKV according to different studies.

**Compound**	**Cell line**	**CHIKV strain; MOI**	**EC_**50**_ (μM)**	**CC_**50**_ (μM)(SI)**	**Assay method**	**References**
Ribavirin (RBV)	Vero	Ross C347 strain; MOI = 0.001	341.53		plaque/microscope/trypan blue	(27)
	Vero	vaccine strain 181/clone 25; MOI = 0.0001	408.2	266.5 (SI = 0.65)	Tox: Viral ToxGlo (Promega), Inf: Virus quantification via plaque assay	(25)
	Huh-7	vaccine strain 181/clone 25; MOI = 0.1	10.56	49 (SI = 4.64)	Tox: Viral ToxGlo; Inf: plaque assay	(25)
	A549	vaccine strain 181/clone 25; MOI = 0.1	480.11	205.86 (SI = 0.43)	Tox: Viral ToxGlo; Inf: plaque assay	(25)
	Vero	ECSA clinical isolate; MOI = 2	10.95	n.d.	Tox: MTT; Inf: plaque formation assay, ELISA-like cell-based assay and IFT	(26)
	Vero	vaccine strain 181/clone 25 (NR-13222); MOI = 0.0001	419.43	n.d.	Inf: plaque assay	(28)
	BHK21	CHIKV-0708 Singapore not mutated; MOI = 1	2.05	n.d.	IFT	(29)
	Huh-7	CHIKV (Asian strain); MOI = 0.1	2.5 ± 0.3	298 ± 22 (SI = 120)	RNA level (RT-PCR)	(24)
	Huh-7	CHIKV (Asian strain); MOI = 0.1	5.5 ± 1.5	298 ± 22 (SI = 54)	Virus titer (yield) by plaque	(24)
	Vero E6	ITA07-RA1; MOI = 0.005	423.6 ± 27.5	>500 (SI > 1.18)	MTS (Promega)	(16)
	Vero E6	LS3; MOI = 0.005	756.8 ± 22.4	>500 (SI > 0.66)	MTS (Promega)	(16)
	Vero E6	LS3-GFP; MOI = 0.005	466.7 ± 38.0	>500 (SI > 1.07)	MTS (Promega)	(16)
	BHK21	ITA07-RA1; MOI = 0.005	20.8 ± 1.1	>500 (SI > 24.04)	MTS (Promega)	(16)
	BHK21	LS3; MOI = 0.005	15.6 ± 1.5	>500 (SI > 32.05)	MTS (Promega)	(16)
	BHK21	LS3-GFP; MOI = 0.005	17.5 ± 1.7	>500 (SI > 28.57)	MTS (Promega)	(16)
Favipiravir (T-705)	Vero	vaccine strain 181/clone 25; MOI = 0.0001	184.53	>6365.4 (SI > 34.5)	Tox: Viral ToxGlo (Promega), Inf: plaque assay	(25)
	Huh-7	vaccine strain 181/clone 25; MOI = 0.1	127.3	>6365.4 (SI > 50)	Tox: Viral ToxGlo; Inf: plaque assay	(25)
	A549	vaccine strain 181/clone 25; MOI = 0.1	245.13	>6365.4 (SI > 25)	Tox: Viral ToxGlo; Inf: plaque assay	(25)
	Vero A	Indian Ocean 899; MOI n.s.	60 ± 10	>636 (SI > 10.6)	MTS (Promega)	(31)
	Vero A	LR2006-OPY1; MOI = 0.1	25 ± 1	>636 (SI > 25.44)	MTS (Promega)	(31)
	Vero A	Italy 2008 (clin.); MOI = 0.1	16 ± 6	>636 (SI > 39.75)	MTS (Promega)	(31)
Sofosbuvir	Huh-7	CHIKV Asian strain; MOI = 0.1	1.0 ± 0.1	402 ± 32 (SI = 402)	Inf: RNA level (RT-PCR); Tox: XTT and PMS	(24)
	Huh-7	CHIKV Asian strain; MOI = 0.1	2.7 ± 0.5	402 ± 32 (SI = 149)	Inf: Virus titer (yield) by plaque; Tox: XTT and PMS	(24)
	Stem cells derived astrocytes (iPSCs)	CHIKV Asian strain; MOI = 1	17 ± 5	n.d.	Virus titer (yield) by plaque	(24)
Mycophenolic acid (MPA)	Huh-7	CHIKV Asian strain; MOI = 0.1	0.8 ± 0.05	370 ± 55 (SI = 463)	Inf: RNA level (RT-PCR); Tox: XTT and PMS assay	(24)
	Huh-7	CHIKV Asian strain; MOI = 0.1	1.1 ± 0.2	370 ± 55 (SI = 336)	Inf: Virus titer (yield) by plaque; Tox: XTT and PMS	(24)
	Huh-7	recombinant CHIKV-118- GFP; MOI = 0.5	1.6	> 100 (SI >62)	Resazurin reduction assay	(32)
	Vero E6	ITA07-RA1; MOI = 0.005	0.6 ± 0.03	>50 (SI > 83.3)	MTS (Promega)	(16)
	Vero E6	LS3; MOI = 0.005	0.6 ± 0.01	>50 (SI > 83.3)	MTS (Promega)	(16)
	Vero E6	recombinant LS3-GFP; MOI = 0.005	0.5 ± 0.07	>50 (SI > 100)	MTS (Promega)	(16)
Prostratin	Vero	CHIKV Indian Ocean strain 899; MOI n.s.	2.7 ± 1.2	~60 (SI~22.8)	MTS (Promega)	(11)
	BGM	CHIKV Indian Ocean strain 899; MOI = 0.001	8 ± 1.2	>100 (SI > 12.5)	MTS/PMS (Promega)	(33)
	BGM	CHIKV Indian Ocean strain 899; MOI = 0.001	7.6 ± 1.3	>100 (SI > 13.16)	qRT-PCR	(33)
	BGM	CHIKV Indian Ocean strain 899; MOI = 0.001	7.1 ± 0.6	>100 (SI > 14.08)	titration assay	(33)
	human skin fibroblasts CRL-2522	Singapore (SGP011), Caribbean strain (CNR20235) + Reunion Island strain (LR2006 OPY1); MOI = 1	0.2-0.5	50 (SI = 100–250)	luciferase assay, qRT-PCR + titration assay	(33)
Chloroquine	Vero	CHIKV Indian Ocean strain 899	10–11	89–100 (SI = 8-9)	CPE reduction, RT-qPCR, MTS (Promega)	(11, 12, 15, 17)
	Vero E6	ITA07-RA1 MOI = 0.005	7.4 ± 1.1	>36(SI > 4.86)	MTS (Promega)	(16)
	Vero E6	LS3 MOI = 0.005	10.6 ± 1.6	>36(SI > 3.4)	MTS (Promega)	(16)
	Vero E6	LS3-GFP MOI = 0.005	5.0 ± 1.7	>36(SI > 7.2)	MTS (Promega)	(16)

*The above mentioned compounds have been repeatedly used in in vitro studies as references. Compound efficacy may considerably between the different studies, depending on the cell line, virus strain, and assay method that is being used. The table aims to give an orientation at what range a control compound might be effective against CHIKV in different cell lines and assay methods*.

*A549, human lung carcinoma cells; BGM, buffalo green monkey kidney cells; BHK, baby hamster kidney cells; CC50, cytotoxicity concentration 50%; CHIKV, Chikungunya virus; CPE, cytopathic effect; EC50, half maximal effective concentration; ECSA, East/Central/South African strain; GFP, green fluorescent protein; Huh, Human hepatocarcinoma cells; IFT, immunofluorescence test/staining; Inf, infection assay; MOI, multiplicity of infection; MTT/MTS, 3-(4,5-dimethylthiazol 2-yl)-2,5-diphenyltetrazolium bromide (salt) assay; n.d., not determined; n.s., not stated; RT-PCR, reverse transcriptase polymerase chain reaction; SI, selectivity index ( = CC50/EC50); Tox, toxicity assay; Vero, African green monkey kidney cells*.

Despite the promising results chloroquine displays in *in vitro* studies, clinical trials with the drug failed to prove any benefit for the patient. Trials for prophylaxis or treatment of CHIKV infection either in macaque models or human patients could not demonstrate advantage of chloroquine over meloxicam (an NSAID) administration (46, 47). The discrepancy between *in vitro* and *in vivo* effectiveness of chloroquine has been described before.

##### Epigallocatechin Gallate (Green Tea Component)

Epigallocatechin gallate (EGCG) is an active polyphenolic catechin and the essential element of green tea (*Camellia sinensis*) extract. Various independent research groups discovered the antiviral properties of EGCG against a number of viruses and recent studies revealed that EGCG also inhibits CHIKV replication *in vitro*. Weber et al. (48) demonstrated that EGCG inhibits CHIKV replication in HEK 293T cells by blocking the entry of CHIKV pseudo-particles that carried the CHIKV envelope proteins. Thus, EGCG prevented the attachment of CHIKV to the target cells.

More recently, Lu et al. (49) showed the benefits of synergism in the combination treatment of CHIKV infected U2OS cells (human bone osteosarcoma cells) with EGCG and suramin. Lu tested EGCG combined with suramin against the CHIKV strain S27 and two clinical isolates. Besides the synergistic effect of the two compounds, Lu could confirm that the EGCG inhibits virus entry, replication, progeny yield as well as CPE of CHIKV *in vitro*.

##### Suramin

Suramin, also known as germanin or Bayer-205, is a symmetrical hexasulfonated naphthylurea compound that has been market-authorized by the U.S. Food and Drug Administration (FDA) for the treatment of trypanosomiasis (trypanosome-caused river blindness, onchocerciasis). The drug acts as a competitive inhibitor of sulphated glycosaminoglycans (GAGs) and heparin. As a number of viruses attach to cells via GAGs, suramin may consequently have anti-viral activity by inhibiting virus entry. The drug proved effective against a number of viruses, including DENV and Venezuelan equine encephalitis virus (VEEV) (50, 51). Against CHIKV, suramin proved effective in various *in vitro* studies (18, 52, 53). Suramin diminished CPE, virus replication and yield in a dose-dependent manner. Ho et al. (18) demonstrated that suramin was broadly effective *in vitro* against various CHIKV strains (Table 1). Ho used BHK-21, U2OS and MRC-5 cells. His group was the first to prove that the compound inhibits entry and transmission of CHIKV through binding onto E1/E2 glycoproteins. Furthermore, they showed that CHIKV infection was hampered in early stages. Virus binding and fusion was disrupted by the binding of suramin with viral glycoproteins. The compound also interfered with virus release. According to their research the EC_50_ of suramin for the inhibition of CHIKV *in vitro* (EC_50_ of 8.8–62.1 μM) is well within the range of non-toxic serum concentrations in humans (70 μM) when treated for river blindness (54).

Henß et al. (53) were also able to verify that suramin blocks CHIKV at early stages of the infection. Furthermore, her group tested the compound successfully against Ebola virus. All her tests were done *in vitro* (HEK 293T, MCF7, and Huh-7 cells). According to Henß however, the drug's side effects on the patient (nausea, vomiting, reversible urticarial rash, kidney damage, and exfoliative dermatitis; furthermore, suramin is connected to hepatic and bone marrow toxicity) might make suramin inappropriate for the treatment of CHIKV infections, a rather mild disease compared to Ebola. To avoid these side effects, Hwu et al. (19) chemically modified suramin and used 20 new conjugated compounds in a CPE screening assay against CHIKV. He identified six compounds with promising activity against CHIKV.

#### Inhibitors of Viral Genome Replication and Translation

##### RNA Interference (RNAi) Targeting CHIKV Genes

Small interfering RNA (siRNA) is able to regulate gene expression by the cleavage of the corresponding messenger RNA (mRNA) (55). The most commonly understood effect of this mechanism is the inhibition of the protein synthesis of certain genes because the mRNA is no longer available. This is referred to as “gene silencing.” The discovery that siRNA is able to inhibit specific genes has led to a vast interest in this particular field. SiRNA was hoped to be used as a potential therapy for the treatment of genetic disorders, cancer, viruses, and other diseases. Bitko and Barik (56) showed that RNA interference (RNAi) was able to inhibit a negative-strand RNA virus.

Since RNAi is an endogenous biological process, potentially every gene can be supressed. In addition to that, siRNAs are easier to identify, synthesize and produce on a large scale than traditional drugs (57). Multiple studies have been conducted to test the possible efficacy of siRNA against viruses *in vitro* and *in vivo* (mice, guinea pigs, macaques and humans) (58). There are two approaches for recruiting RNA interference as antivirals: (1) targeting specific viral sequences; (2) targeting the host cell.

(1) Targeting specific viral sequences with synthetic siRNA:

SiRNA can be created in the laboratory and preferably targets conserved regions. Theoretically any specific viral gene can be disabled. This is an advantage over classical small drug molecules that have to be fitted to a target protein which usually is only present at certain sites in the cell (59).

Dash et al. (60) designed and evaluated siRNA sequences targeting CHIKV nsP3 and E1 genes in Vero cells. They could demonstrate that these siRNAs curbed CHIKV titres by 99.6% in siRNA transfected cells 24 h after infection. However, this reduction could not be sustained at 72 h, possibly because of the intracellular degradation of the siRNA. In 2013, Parashar et al. conducted *in vitro* studies in Vero-E6 cells, where he used siRNAs targeting nsP1 and/or E2 mRNA. He succeeded in downregulation of CHIKV replication for more than 90%. *In vivo* studies in CHIKV-infected Swiss albino and C57 BL/6 mice showed a complete inhibition of CHIKV replication when these siRNAs were administered 72 h post-infection (61). Lam et al. (62) could also demonstrate that CHIKV infection could effectively be supressed in the mouse model when pre-treating the animals with (small hairpin) shRNA (a precursor form of siRNA) against CHIKV E1 and nsP1 (62).

More recently, due to its advantages over siRNA and shRNA as far as stability, effectiveness, and toxicity are concerned, the artificial miRNA (amiRNA) based approach is in the focus of research. Bhomia et al. (63) showed the effectiveness of amiRNA for inhibition of Venezuelan equine encephalitis virus (VEEV). Saha et al. (64) successfully tested vector-delivered amiRNA against CHIKV infected Vero cells and efficiently inhibited CHIKV replication. One problem arising from this approach is the development of resistant mutants. A possible solution might be a combination therapy with a cocktail of various siRNAs.

(2) Targeting the host cell with siRNA:

It is also possible to target mRNAs for cellular accessory or entry proteins so that they can no longer be used by the virus during infection. Researchers tried to use the mutationally more stable host proteins as targets instead of the rapidly mutating viral proteins (58).

Rathore et al. were able to show in 2014 that by silencing the heat shock protein 90 (Hsp90) transcripts with siRNA, CHIKV replication is interrupted in cultured cells. Heat shock protein 90 (Hsp90) is known to play a key role in the replication of CHIKV and other viruses and is a highly abundant molecular chaperone (65). Rathore found out that Hsp90 interacts with the nsP3 and nsP4 proteins of CHIKV to promote virus replication (66). For further “Host-targeting Antivirals” (see section Antivirals Against Chikungunya Virus).

Both siRNA approaches (viral or host target approach) share the same issues in bioavailability, delivery, and specificity. siRNA is not very stable. It is rapidly degraded in the cell/organism. Furthermore, when systemically applied, siRNA has to reach the target cells. Effective pharmacological use of siRNA requires “carriers” that deliver the siRNA to its intended site of action. siRNA displays poor cellular uptake and is not able to pass through the blood-brain-barrier (67). Small hairpin RNAs (shRNAs) present a solution to some of these flaws. shRNAs are ~70 nt long precursor siRNAs that are introduced into the cell by viral or bacterial vectors (e.g., plasmids). After expression in the nucleus, the shRNA is being transported to the cytoplasm where it is further processed by Dicer proteins. It is subsequently loaded into the RISC for specific gene silencing activity in the same manner as synthetic siRNAs (68).

siRNA often turns out to be unspecific. The suppression of other genes (the so-called “off target effects”) may lead to unknown consequences due to dangerous mutations and unwanted gene expression (69). SiRNA may also interfere with the host immune response (70). Consequently, the long-term safety of si/shRNA treatment is yet unclear as there are only few *in vivo* RNAi long-term studies (58).

##### Inhibitors of CHIKV nsP1

The non-structural protein 1 (nsP1) is a palmitoylated protein with methytransferase (MTase) and guanylyl transferase (GTase) activity. The protein consists of 535 amino acid residues and is responsible for the capping and the methylation of the newly synthesized viral and genomic RNAs (39). The added cap structure on the viral mRNA ensures the translation of the RNA and prevents its degradation from cellular 5′-endonucleases. On its N-terminal domain, the nsP1 has a α-helical amphipathic loop as well as a palmitoylation, which both act as anchors to attach the nsP1 and the nsP1-containing polyproteins/replication complex (RC) to the host's cellular membrane (71). Various studies could show that the palmitoylation of nsP1 is an important feature for the replication of some alphaviruses (72, 73). Depalmoylated Semliki Forest virus (SFV) mutants displayed a diminished pathogenesis in mice (72). Likewise, Zhang and colleagues (74) demonstrated *in vitro* that by inhibiting the enzyme responsible for the palmitoylation of proteins during CHIKV infection, CHIKV replication could be suppressed. There is evidence suggesting that nsP1 has additional functions during alphavirus infections like the development of cell filopodia and the rearrangement of actin filaments (73). Especially the MTase and GTase-like activities of nsP1 present a viable target for antiviral compounds since both enzymatic properties are essential for virus replication. The GT activity of nsP1 is dependent on successful MTase activity (75). Interestingly, unlike cellular MTase and GTase enzymes, the nsP1 does not contain canonical signature motifs and the mechanism of the enzymatic action differs from the cellular cap formation. Thus, there is the possibility of identifying molecules that selectively inhibit viral nsP1 without affecting the host cell capping enzymes' activity (76). Compared to the other nsPs, the research on antivirals that target nsP1 has been poor. Lampio et al. tested 50 guanosine/cap analogs for their activity of inhibiting SFV nsP1 20 years ago (77). Recently, Bullard-Feibelman developed an assay to screen and identify possible CHIKV nsP1 inhibitors (78). Two years later, the same research group presented their results on a high throughput screening (HTS) of 3,051 compounds and their successful identification of promising hit compounds like the naturally derived compound “lobaric acid” (Table 1) (20). Gigante et al. found a strong inhibitor of CHIKV replication among a new family of compounds named [1,2,3]triazolo[4,5-d]pyrimidin-7(6H)-ones (Table 1) (21). It was not until 2016 when reverse genetics carried out by Delang et al. could identify the CHIKV nsP1 as the target for this potent compound (79). New derivatives of these compounds also inhibited the GTase activity of CHIKV and VEEV nsP1 (14). A report from Jones et al. (80) postulated that nsP1 was an antagonist of tetherin (an antiviral host factor that helps to retain the viruses at the surface of the infected cells). These findings gave rise to hope that nsP1 could be considered as a target for developing tetherin-mediated therapeutics against CHIKV (80). However, a more recent study on the subject could not confirm Jones' report since no evidence for tetherin-antagonists in alphaviruses was found (81).

##### Inhibitors of CHIKV nsP2

The CHIKV nsP2 has multiple enzymatic activities and thus plays a central role in CHIKV replication. nsP2 has auto-protease activity at its C-terminal end for cleaving the non-structural viral polyprotein (nsP1234) into the individual nsPs. There is a methyltransferase-like region of unknown function. The N-terminal half has terminal helicase, nucleoside triphosphatase (NTPase), and RNA triphosphatase activities (82). The triphosphatases are involved in RNA capping and also fuel the RNA helicase domain with energy. Additionally, CHIKV nsP2 is a virulence factor as it is able to stop the host cells mRNA transcription and translation, thus tampering with the hosts immune response. This is referred to as “transcriptional shut-off” (83). In fact, a recent study was able to show that nsP2 (as well as nsP3) exhibit RNA interference (RNAi) suppressor activity (84). Viral suppressors of the RNAi pathway (VSR) have been found encoded in various viruses (including flaviviruses) before. Yet, the report of Mathur et al. was the first to show VSR in alphaviruses. Moreover, Fros and colleagues found out that CHIKV nsP2 suppresses the type I/II interferon-stimulated JAK/STAT signaling pathway, which consequently inhibits the hosts antiviral response and defense mechanisms (82). It has previously been shown in other viruses that especially the protease function poses an interesting target for antiviral drugs (85).

###### Compounds designed in silico

Marcella Bassetto and colleagues applied a structure-based virtual screening strategy to find possible CHIKV nsP2 inhibitors. The molecules in question have been modeled to potentially fit and thus block the nsP2 protease binding site.

Based on this model, Bassetto performed a virtual screening of ~5 million compounds and investigated the structure-activity relationship of the identified hits. After a final visual inspection, 15 derivates were selected to be potential CHIKV nsP2 inhibitors. As only 9 were commercially available, those were evaluated in a virus-cell-based CPE reduction assay. Compound 1 performed best and was predicted to fit the central portion of the nsP2 protease active site (Table 1). The compounds' ability to act as a selective CHIKV replication inhibitor was then further investigated by performing a virus yield assay on Vero cells. The assay confirmed the findings of the CPE reduction assay.

Furthermore, Bassetto created structural analogs of Compound 1 and tried to chemically optimize the properties of the compounds. She designed and synthesized two new derivates with one showing a slightly better antiviral activity profile than compound 1. With her work Bassetto proved that a combination of molecular modeling with different *in silico* techniques and classical medical chemistry methods can lead to the discovery of novel and selective antiviral compounds.

Jadav et al. (86) tested a series of derivates of 1,3-thiazolidin-4-ones for their antiviral activity in a CPE reduction assay on Vero cells. Five compounds showed promising CHIKV inhibition properties. The authors assumed the mode of action may be that of protease inhibition, after they carried out molecular docking simulation with the available X-ray crystal structure of the CHIKV nsP2 protease (86). Here, the computer-aided binding model was used to explain possible mechanism of action, while Bassetto used the docking simulation to model compounds accordingly. Still, neither of these studies actually tested the ability of the predicted compounds to inhibit the protease activity of CHIKV nsP2.

It was the group of Das that actually designed and tested 12 compounds specifically on their ability to block the nsP2 (22). The researchers managed to create a test to validate whether the compounds actually inhibit nsP2. Das designed the compounds specifically to fit the nsP2 active site, using the same method as Bassetto and employing Compound 1 of Bassetto as a template for his products.

The group then systematically analyzed the ability of the compounds to inhibit the protease activity of the purified enzyme in cell-free assays. Two different cell free assays were employed, one being an end-point assay, the second one being continuous. In the end point assay, Das used full-length recombinant CHIKV nsP2 as the protease and a recombinant protein substrate containing the nsP2 cleavage site that was located between enhanced green fluorescent protein (EGFP) and thioredoxin. If the nsP2 was fully functional, the protein substrate was being processed, making it possible to detect the products by separating them by SDS-PAGE and visualizing the results with a Coomassie blue staining. The method on how to express and purify the recombinant proteins has been described earlier by the same group (87).

To verify his finding, Das additionally used a fluorescence resonance energy transfer (FRET)-based assay to compare the efficiencies of different inhibitors. This kind of assay had originally been described for the HIV protease by Matayoshi et al. (88). It is a continuous assay that makes it possible to collect information on the initial period of the reaction. In Das' assay, the nsP2 protease processed a peptide substrate with the nsP3/nsP4 cleavage site of CHIKV P1234 polyprotein (89). The substrate had a quencher at the N terminus and a fluorescent molecule at the C terminus. Cleavage of the substrate by nsP2 protease results in fluorescence that can be detected at an emission wavelength of 490 nm.

With these assays, Das managed to show that the majority of his compounds inhibited the nsP2s ability to process recombinant protein and synthetic peptide substrates. He also discovered that the original template molecule from Bassetto performed very poorly as a specific nsP2 inhibitor in these cell free assays, despite the fact that it had an EC_50_ of ~5 μM in cell-based assays against CHIKV (12). Das then tested his compounds successfully in cell-based assays against CHIKV. The fact that some compounds did not inhibit the CHIKV nsP2 protease function in the cell free assays and yet managed to curb CHIKV infection in cell-based assays suggests that the antiviral activity of these compounds may be at least in part due to other mechanisms than the inhibition of protease activity of nsP2 (Table 1) (22).

###### Compounds inhibiting the nsP2 mediated “transcriptional shut-off”

Lucas-Hourani et al. (23) developed a phenotypic cell-based functional assay to detect CHIKV nsP2 protease inhibitors. In particular, compounds that inhibited the nsP2 mediated “transcriptional shut-off” mechanism were to be detected. As mentioned before, the nsP2 protease is able to bind to cellular transcription factors and thus induce downregulation of the cell's immune response. In Lucas-Houranis' assay luciferase expression is induced when the cellular functions are working at a normal level. If nsP2 protease is blocked by antivirals, the cells mRNA transcription is properly restored and thus a replication of luciferase takes place, resulting in an increased signal.

The assay is thus based on a recombinant human cell line (HEK-293T) that expresses CHIKV nsP2 together with various reporter gene constructs (on three plasmids). Lucas-Hourani used this transfected cell line to establish an assay suitable for screening compounds for their nsP2 inhibition activity. From a pool of 3,040 molecules, he detected one with no toxicity that particularly blocked nsP2 activity *in vitro* (Table 1) (23).

##### Inhibitors of CHIKV nsP4 and Viral Genome Replication

The nsP4 is the sole protein with a polymerase function and is responsible for the RNA synthesis of the (replication complexes) RCs. The ~100 residues at the N-terminal region are specific to alphaviruses. The nsP4 has ~70 kDa and contains the core RNA-dependent RNA polymerase (RdRp) domain at its C-terminal end. The structure of the RdRp is typical and encompasses fingers, palm containing the GDD motif at the active site and thumb domains (90). The RdRp is able to copy the genome into a complementary minus-strand which is in turn copied into genomic and subgenomic RNAs by the polymerase with the help of the other viral nsPs in the RC. Mutation studies revealed a TATase (tyrosine aminotransferase) activity in the RdRp domain. Thus, the nsP4 may be generating the poly(A) tail at the 3'terminal of the genome (91). For more details on the nsP4s role during genome replication and its fundamental function I refer to the review of Pietila et al. (9).

Research has recently focussed on finding antiviral compounds against viruses of the *Flaviviridae* family [hepatitis C virus (HCV), Zika, Dengue, Yellow Fever virus (YFV), tick borne encephalitis virus (TBEV)], most of which are arboviruses. Especially Zika and Dengue can cause coinfections with CHIKV and the initial symptoms of the three diseases look very similar. Since the diagnosis is costly and time consuming, it is crucial to find a pan-antiviral that works against all of them. All three viruses are +ssRNA viruses and there is a reasonable chance that they share conserved motifs in the orthologous RdRp enzyme (24, 91). The remarkable homology of the nsP4 among the alphaviruses makes it possible that antivirals blocking the nsP4 may exhibit their activity over a broad spectrum of viruses. With human cells lacking this specific polymerase the chances of adverse side effects of RdRp inhibitors are minimized (92).

###### Nucleoside analogs and proTides

Nucleoside analogs (NAs) are synthetic, chemically modified nucleosides consisting of a sugar and a nucleic acid analog. Nucleotide analogs additionally have one to three phosphate groups attached to the 5′-site. In the cell, they are processed the same way as the natural (endogenous) nucleosides. After their uptake into the cell and their metabolization, the NAs can act on cellular functions. They mimic their physiological counterparts and block cellular division or viral replication by impairing DNA/RNA synthesis (they usually cause termination of the nascent DNA/RNA chain) or by inhibition of cellular or viral enzymes involved in the nucleoside/tide metabolism (93, 94). The FDA has approved more than 25 nucleoside analog drugs used for the therapy of viral infections such as HIV/AIDS (tenofovir), hepatitis B (lamivudine/entecavir), and C (sofosbuvir) or herpes (acyclovir) (93, 95). Besides being antiviral agents, NA drugs are also applied in the therapy of cancer, rheumatologic diseases and even bacterial infections (96).

Before NAs can actually work as antivirals, they have to be phosphorylated in the host organism. Three consecutive phosphorylation reactions are necessary to activate the prodrug. The first reaction to the 5′-monophosphate is usually a rate-limiting step, which also means that if this first phosphorylation does not take place, the drugs remains inactive (97). This might happen either because the virus does not induce a specific kinase or has acquired a mutation in this particular enzyme resulting in resistance to the compound because the host cell is not able to phosphorylate the NA.

Monophosphate NAs have come into focus in order to avoid this problem and improve the therapeutic properties. However, these phosphate analogs (possessing a CO–P bond) proved to be prone to esterase and phosphatase hydrolysis. As an alternative, chemists investigated replacing the phosphate group by an isosteric and isoelectronic phosphonate moiety (CH2-P bond). This led to the discovery of nucleoside phosphonate analogs (NPs), which are chemically and enzymatically more stable than the phosphate analogs (98).

Toxicity and side effects of nucleoside/-tide analog drugs often result from their off-target use by host polymerases and their incorporation into RNA or DNA. The observed toxicities tend to be highly unpredictable and even closely related analogs may prove toxic for different organs (95). Various mechanisms for NAs toxicity have been discovered, the most characteristic is due to their affinity to host mitochondrial gamma polymerase (99). The NAs enter the mitochondria and are either incorporated into the mitochondrial DNA or block its synthesis.

Since NAs, nucleoside 5′-monophosphates or 5′-phosphonates are charged molecules and penetrate the cell membrane very poorly, they are not suited for oral administration. Research tried to improve the pharmacological properties and bioavailability of this class of compounds. This led to the discovery of the ProTides approach by McGuigan in 1998 (100, 101). The researchers designed a novel prodrug in which the phosphate was chemically protected or masked. This group of prodrugs became known as “ProTides” (pronucleotide) and as a result from the masked phosphate, this construct is able to pass the cell membrane via facilitated passive diffusion (94).

In the cell, the ProTide is enzymatically cleaved, thus releasing the masking groups from the nucleoside monophosphate/phosphonate which can be further transformed into the active 5′-triphosphate form of the NA. Various natural and unnatural amino acids can serve as the masking amino acid motif. All ProTide drugs that have reached the clinic, feature l-alanine (94). With the prodrug strategy, medical chemists were able to solve the main pharmacological problems associated with NAs, namely poor cellular uptake and poor metabolism into their phosphorylated forms.

###### Ribavirin

Ever since its discovery in 1972, ribavirin (1-β-D-ribofuranosyl-1,2,4-triazole-3-carboxamide, also known as Virazole), a synthetic guanosine nucleoside analog, has been used as a compound against various viruses (102).

Ribavirin (RBV) is one of few FDA approved antiviral drugs in clinical use that is effective against respiratory syncytial virus in infants and chronic hepatitis C virus infections in combination with pegylated interferon (IFN)-α (103, 104). Apart from the FDA approved indications, RBV has shown efficacy against a variety of virus infections including haemorrhagic fever and measles (105, 106). Huggins and colleagues could also prove RBV's effectiveness against viruses of the alphavirus family *in vitro* (107). Multiple studies confirmed his findings by testing RBV *in vitro* against CHIKV either as a monotherapy (25) or in combination with doxycycline (26) or IFN-α (27, 28). Especially, Franco et al. (25) demonstrate that the effectiveness of antiviral agents against CHIKV differs considerably between host cell lines (Table 2).

Various different mechanisms of action have been attributed to RBV which might explain its broad-spectrum antiviral activity. The major mechanism, by which the replication of RNA viruses is being inhibited, is curbing the cellular guanosine triphosphate (GTP) pools by blocking the inosine monophosphate dehydrogenase (IMPDH) (108). Another indirect mechanism is the immunomodulation of the host's adaptive immune response: RBV triggers a suppression of the T-helper type 2 response and an induction of the T-helper type 1 response (109). The type 1 response is responsible for an increased clearance of infected cells. Additionally, RBV is believed to directly inhibit RNA capping. Other findings suggested that RBV interferes with the guanylyl transferase and/or methyltransferase activity of the nsP1, leading to a production of mRNAs that are not fit for translation (110). RBV is said to directly inhibit the viral polymerases, thus hampering the virus' genome replication (111). This has also been proposed by other studies that suggested RBV to directly inhibits nsP4 RdRp by interacting with its Cys483 residue, resulting in a decrease in replication fidelity (112). This would confirm the theory that RBV leads to error catastrophe via increased mutation frequency (nucleotide transitions) because of the incorporation of ribavirin triphosphate (RTP) into the newly synthesized viral genomes (113). Others found indications that RBV promotes IFN signaling by modulating specific genes and thus potentiating IFN action (114).

RBV, albeit a success as a broad-spectrum antiviral *in vitro*, has rarely been reported to be the subject of *in vivo* trials against CHIKV in humans. Ravichandran and Manian (115) treated 10 patients with confirmed CHIKV infection. Before treatment the infection had not been resolved after 2 weeks and resulted in crippling lower limb pains and arthritis. The patients were treated with 200 mg RBV twice daily for 7 days. A control group of 10 similar patients was only given analgesics when required. According to Ravichandran and Manian the patients of the RBV group showed a significant improvement in the joint pains and 8 patients out of 10 had a reduction in tissue swelling. Ravichandran concluded that RBV may indeed have a direct antiviral property against CHIKV infection and might lead to a faster recovery of the patients. However, the study had some flaws: (1) only a small number of patients were considered; (2) the study was not a randomized controlled study (a so-called double-blind study) where the RBV group was compared with a group receiving placebo; (3) the drug was administered in the subacute phase of the disease, thus some of the improvement could be attributed to a normal course of healing. A recent *in vitro* study of Mishra et al. (116) suggested that RBV is only effective in the earlier stages of the CHIKV lifecycle; the benefit of giving the drug in a subacute or chronic phase might therefore be questioned.

The doses at which RBV would have to be administered in order to reach its full potential as an antiviral *in vivo* are associated with severe side effects such as haemolytic anemia, pulmonary, dermatologic, and teratogenic effects and can thus only be justified if the infection is life-threatening (117).

RBV's success as an antiviral is probably attributed to its ability to act simultaneously via multiple mechanisms. Usually, when an antiviral interacts at various cellular and viral processes, the chances for drug resistant mutants are diminished. But, in case of RBV, various resistant viruses have been reported, such as Sindbis virus, Hepatitis C Virus and CHIKV, showing yet again, how quickly viruses are able to adapt (52, 110, 118). Taking these developments into account, RBV might still be interesting as a component in an antiviral “cocktail” consisting of multiple drugs with various modes of action, where the dosages of the drugs themselves could be reduced due to synergism and the risk of adverse effects could thus be minimized.

###### β-d-N4-hydroxycytidine (NHC)

A report from Ehteshami et al. (30) stated the outcome of experiments dealing with β-d-N4-hydroxycytidine (NHC), another modified NA. NHC was identified to successfully inhibit CHIKV replication in different replicon cell lines as well as in infectious models *in vitro* (Table 1). One year later, another group published that NHC was able to curb the release of genome RNA-containing VEE virions and their infectivity in *in vitro* test with Vero cells (119). This discovery supports the idea that the polymerase activity of the nsP4 is quite conserved and that drugs targeting this particular activity might show efficacy against various alphaviruses. The antiviral activities of NHC are probably due to the compound acting as a pyrimidine analog that may directly target the viral polymerase and cause chain-termination. Alternatively, the compound might induce accumulation of mutations in virus-specific RNAs which are either lethal or lead to viral genomes that are incapable of replication (30). Urakova suspects a dual effect of NHC on VEEV by causing a modest decrease *in virion* release and a strong decrease *in virion* infectivity. This idea supports the theory that mutations caused during the replication process lead to “error catastrophe” or “lethal defection” (119, 120).

Urakova reported that NHC only triggered the development of a low-level resistance in VEEV against NHC, which makes it a very promising compound that might substitute RBV. These findings are very encouraging. Nevertheless, further studies with more relevant human cell lines, animal models as well as other viruses are needed to confirm whether this compound has a future as a broad-spectrum antiviral.

###### Favipiravir (T-705) and its defluorinated analog (T-1105)

Favipiravir (T-705, 6-fluoro-3-hydroxy-2-pyrazinecarboxamide) is an approved drug in Japan for the treatment of influenza virus infections (121, 122). The drug is a purine analog and functions as a broad-spectrum antiviral agent which has also been reported to inhibit (*in vitro* and *in vivo*) the replication of a number of RNA viruses such as arenaviruses, bunyaviruses (123) and alphaviruses (124–126). During the 2014/2015 Ebola epidemic in western Africa, T-705 proved beneficial for infected patients (127).

Favipiravir is a prodrug, which is phosphoribosylated in the cell into its active form, a ribofuranosyl 5′-triphosphate metabolite (favipiravir-RTP). It acts as a pseudo purine and inhibits the viral replication of influenza. Two modes of action have been suggested: There is evidence that favirpiravir-RTP specifically blocks the influenza virus RNA-dependent RNA polymerase (RdRp) by binding at certain domains of the enzyme (122). Others suggested that favipiravir-RTP is incorporated into the nascent viral RNA, thus leading to lethal mutagenesis or preventing further extension of the RNA strand entirely by chain termination (128, 129).

As favipiravir is relatively novel, the information on its *in vitro* efficacy is limited. Values vary depending on the assay, cell line and virus strain used (Tables 1, 2). Apart from favipiravir itself, the defluorinated analog T-1105 has worked as an antiviral drug against CHIKV in *in vivo* experiments with mice (31). The drug prevented mice from developing severe neurological disease and reduced the mortality rate of the CHIKV infected animals. A dosage of 300 mg/kg T-705 daily and orally proved especially beneficial for CHIKV infected mice during the acute phase of the disease (125). Delang also identified T-705 resistant CHIKV variants *in vitro*. The mutant had acquired a mutation in the motif F1 of the RdRp, which seems to be important in the nucleoside triphosphate binding during and in the initiation of the viral RNA synthesis of +ssRNA viruses (130). Yet, Abdelnabi et al. (126) suggest that favipiravir has a high barrier of resistance. Abdelnabi made experiments in which he tried to create T-705-resistant coxsackievirus B3 (CVB3) (another +ssRNA virus), by point-mutating the same F1 motif. These efforts resulted in either low-fidelity RdRp or unviable virus. Since NTP binding is a major fidelity checkpoint, point mutations in this F1 motif could destroy the activity of the polymerase or reduce catalysis (131).

The fact that resistant mutants develop, demonstrates how quickly RNA viruses can adapt to selective pressure via mutations. Understanding the role of conserved motifs like F1 is of great importance in order to understand the mode of action of certain drugs and possibly design more potent compounds.

###### Sofosbuvir

Sofosbuvir (β-D-2′-deoxy-2′-α-fluoro-2′-β-C-methyluridine, formerly known as PS-7977 or GS-7977) is a RdRp inhibitor approved by the FDA for the treatment of HCV infections (132). The drug is a nucleotide analog that is orally available and functions as a prodrug. In hepatocytes, sofosbuvir is metabolized to 2′-F-2′-C-methyluridine monophosphate (UMP) and further phosphorylated into its active triphosphate form (UTP). During the viral genome synthesis, UTP functions as a chain terminator, thus inhibiting HCV replication and production at the site of infection, in this case the liver (133). Sofosbuvir has recently been reported to inhibit YFV and ZIKV replication *in vitro* and *in vivo* (134–136).

Sofosbuvir has been tested against CHIKV *in vitro* (Huh-7 cells and astrocytes) and *in vivo* (mice) (24). The drug inhibited CHIKV replication and was three times more potent in inhibiting CHIKV in human hepatoma cells than RBV (Table 2). In human induced pluripotent stem cell-derived astrocytes, sofosbuvir did impair virus production and cell death in a MOI-dependent manner, yet not to such a degree as in the Huh-7 cells. This may be due to the fact that hepatocytes have the most effective system of turning the prodrug sofosbuvir into its active form (UTP), whereas astrocytes show less metabolic activity in this respect and thus have less of the active UTP form of the drug available (133). Furthermore, sofosbuvir prevented CHIKV-induced arthralgia-related paw oedema in adult mice as well as mortality in neonate mice (24). Since CHIKF can lead to chronic arthralgia, further studies are needed to evaluate if sofosbuvir in a combination therapy alongside anti-inflammatory drugs is beneficial to patients suffering from chronic CHIKV associated arthritis.

Interestingly, humans tolerate the drug better than mice. A 400 mg daily dose over a period of 12–24 weeks is the standard therapy for HCV patients (133), while doses of >33 mg/kg/day in a 7 day regime proved to be toxic to mice (136). The reason for this observation might be the decreased stability of sofosbuvir in rodent serum. This raises the question of how significant rodent models are for the evaluation of sofosbuvir or whether other (animal) models might be more representative.

Similar to favipiravir, sofosbuvir resistant HCV strains have been reported (137). Yet, the development of sofosbuvir resistant mutants seems to be slower compared to HCV inhibitors targeting other proteins. Researchers hold the high degree of amino acid conservation within the RdRp domain as well as the lack of fitness in mutated viruses responsible for this phenomenon (136).

Nevertheless, the fact that sofosbuvir blocks the viral replication of CHIKV as well as several flaviviruses is strong evidence for the presence of conserved motifs among RNA polymerases from +ssRNA viruses. The recent advances in elucidating the nsP4 structure and core domain function of CHIKV highlight these observations and may confirm that the RdRp is a feasible target for pan-antiviral molecules (91).

##### Other Viral Genome Replication Inhibitors

###### Mycophenolic acid (MPA)

Mycophenolic acid (MPA) had already been discovered in 1893 and was isolated in 1896 as an antibacterial molecule produced by *Penicillium brevicompactum* (138). MPA is licensed by the FDA as a drug for transplantation rejection (139). The drug inhibits cellular inosine monophosphate dehydrogenase (IMPDH) and thus decreases the intracellular pools of guanosine triphosphate (GTP) and 2′-deoxyguanosine triphosphate (dGTP). This causes a disruption of viral and cellular RNA, DNA, and protein synthesis (140). Two derivatives of MPA are available for clinical use: mycophenolate mofetil (MMF, CellCept) and mycophenolate sodium (MPS, Myfortic). Mycophenolate mofetil is the orally bioavailable prodrug form of MPA. MPA has shown antiviral activity against DENV and Orthopoxvirus (141, 142).

Although MPA was reported to inhibit CHIKV *in vitro* in 2011, tests done in 2018 could not confirm these findings (143, 144). However, Ferreira tested MPA as a control alongside his compounds and indeed received good EC_50_ values, with MPA even performing slightly better than sofosbuvir and with a much better selectivity index (SI = CC_50_/IC_50_) than RBV (24). Likewise, other research groups used MPA as a reference against CHIKV and evaluated the efficacy against CHIKV (Table 2) (16, 32).

There are various studies confirming the antiviral, antibacterial, antifungal, immunosuppressive, and anticancer properties of MPA or its derivates (145). Yet it is important to deliberate whether the benefits of MPA as an antiviral outweigh its adverse effects as an immunosuppressant.

##### NsP3 and Possible Inhibitors

The nsP3 consists of three domains. The N-terminus has a macrodomain, while the C-terminus holds a hypervariable domain (HVD). The central part of the protein contains a zinc-binding domain which is sometimes referred to as the alphavirus-unique domain (AUD), a region that shares a strong sequence homology across the alphaviruses. The role of the AUD is so far undefined but the domain seems to be important in RNA replication and in the synthesis of negative sense and sub-genomic RNA (146).

There are hints indicating that the nsP3 is involved in inhibiting the assembly of the host cells stress granules (SG) which are essential for the degradation of viral mRNA (147). NsP3 is usually found in complex with other nsPs during infection. It also interacts with host factors. Saul et al. (148) discovered that the amount of nsP4 increased in a recombinant SFV with a duplicated nsP3-encoding sequence. Saul concluded that nsP3 is involved in the stabilization of nsP4. He could furthermore back other studies' findings that nsP3 is important for the (neuro-) virulence of old-world alphaviruses (148). In New-World alphaviruses, neurovirulence is mainly determined by structural proteins, particularly E2 (149).

So far, the complete function of the nsP3 macrodomain has not been fully unraveled although its crystal structure has been known since 2009 (PDB id: 3GPG and 3GPO) (150). The N-terminal macrodomain is highly conserved among alphaviruses but also occurs in other positive-strand RNA viruses such as coronaviruses and hepatitis E virus (151). There is evidence that the viral macrodomains bind ADP-ribose, dephosphorylate ADP-ribose-1”-phosphate and act as de-ADP-ribosylating enzymes thus counteracting antiviral ADP-ribosylation (152). Other studies indicated that the most likely biochemical function of viral macrodomains is de-ADP-ribosylation. By enzymatically removing mono- and poly-ADP-ribose from proteins, macrodomains might oppose the host cells' antiviral response (153). Furthermore, the mono(ADP-ribosyl)hydrolase activity of the nsP3 is critical for CHIKV replication in vertebrate hosts and insect vectors, and determines virulence in mice (154). These findings suggest that the macrodomain plays an important part in the host-pathogen conflict.

Nguyen et al. virtually screened a database of 1,541 compounds for possible hits that might block the nsP3 macrodomain of CHIKV (155). The group combined molecular docking, virtual screening, and molecular dynamics simulations to identify potential inhibitors. They ended up with three ligands that might have potential as nsP3 inhibitors. However, these findings were achieved *in silico* and still need to be verified by experimental studies *in vivo*.

Until Varjak et al. discovered a degradation signal at its C-terminus, nsP3 was thought to be a rather stable protein. Varjak could demonstrate that the nsP3 of SFV and Sinbis Virus (SINV) was degraded rapidly when the protein was expressed individually. On the other hand, nsP3 was significantly stabilized when it was expressed in the nsP123 polyprotein form (156). The role of this C-terminal degradation signal is still unknown but there are various hints that it may contribute to granting the optimal stoichiometry of the nsPs.

Especially the HVD at the C-terminal region of the nsP3 seems to be a center for interactions with host cell proteins, including stress granule (SG) components which might help the virus adapt to distinct cellular environments. Data suggests that the HVD interacts with several host factors through a conserved proline (P)-rich and duplicate FGDF motif. The letters of the motif correspond to the according amino acids, two phenylalanine residues which are separated from each other by a glycine and an aspartate residue (157). These interactions are needed for the assembly of virus genome replication complexes (158, 159). The FGDF motif seems particularly important for the successful replication of alphaviruses in mammalian cells. Experiments with CHIKV revealed that the virus' nsP3 has two FGDF motifs that bind to certain domains of the SG components in mammalian cells (160). SGs usually block host and viral translation. The interactions between the CHIKV nsP3 and the SG domains impede the organization of the SGs and thus may allow virus replication (147, 161, 162). When the alphavirus nsP3 HVD is mutated in a way that both FGDF motifs are disrupted, CHIKV is inactivated and SFV as well as SINV are attenuated in mammalian cells. If only one FGDF motif is present in CHIKV or SFV nsP3, the affinity for the SG domains is reduced and the virus is attenuated as well. This leads to the conclusion that alphaviruses need two FGDF motifs for a successful viral replication in mammalian cells (146, 160, 161).

The HVD seems also to be a determinant for virulence in some viruses. There is evidence that the conserved FGDF motifs in the HVD of chikungunya virus nsP3 are required for the effective transmission of the virus from *Aedes aegypti* mosquito saliva to a vertebrate host (163).

The nsP3 seems to be an important protein in determining vector specificity. ONNV, which is closely related to CHIKV, is the only alphavirus known to be transmitted by *Anopheline* mosquito species. CHIKV on the other hand, is mainly transmitted by *Aedes* mosquitoes. Experiments with chimeric CHIKV expressing ONNV nsP3 revealed that *Anopheles gambiae* mosquitoes become susceptible for CHIKV although being naturally immune to WT CHIKV (164). This observation is in line with previous findings suggesting that nsP3 might be involved in specific protein-protein interactions and thus carries out host cell-dependent functions (165). A recent study revealed that nsP3 suppresses RNAi alongside nsP2 in CHIKV infected insect cells (84). As RNAi is an antiviral defense mechanism in various organisms that leads to a degradation of viral RNA, the suppression of RNAi by viral proteins enhances infection.

The impact of these interactions on biological and biochemical processes of the host cell at early stages of the infection are still under heavy investigation. There is hope that the interacting regions might prove valuable targets for intervention and opens new possibilities for vaccine development and antiviral drug discovery.

Kaur et al. (29) reported the discovery of the anti-CHIKV properties of **harringtonine**, a cephalotoxin alkaloid from the *Cephalotaxus harrintonica* trees. It was suggested that the compound inhibits the early stages of CHIKV infection after cellular endocytosis (29). Harringtonine was proposed to interfere with the protein translation of CHIKV since it seemed to inhibit the production of nsP3, E2 proteins, and CHIKV RNA (29, 166). Harringtonine was approved in 2012 by the FDA as a drug for the treatment of chronic myeloid leukemia (167). **Homoharringtonine**, an analog of harringtonine with an additional methyl group, was reported to have anti-CHIKV properties as well. According to Kaur, both compounds display minimal cytotoxicity on BHK-21 cells and primary human skeletal myoblasts at the dosage needed for inhibiting CHIKV. However, the drug itself is labeled as a cytotoxic agent and according to the Globally Harmonized System (GHS) harringtonine is fatal if swallowed (H300), in contact with skin (H310) or if inhaled (330) (168). This may be the reason that although Kaur's original article has been cited repeatedly, no studies on the anti-alphavirus properties of harringtonine have been published in the past 7 years.

### Host-Targeting Antivirals

Many viruses depend on host factors to ensure their replication or are inhibited by such. Host factors present a valuable target for drugs to interfere in the virus' life cycle either by inhibiting host factors on which the virus relies on or by promoting host factors that curb virus infection. Since host factors also play vital roles in normal physiology, their inhibition or promotion can lead to abnormal physiological function and toxicity. The impact such interference may have on the host organism must thus be critically elucidated. Ideally therapeutics would target interactions between host and viral factors without disrupting essential cellular processes. For the interested reader we refer to the review of Wong and Chu (169) that summarizes the current knowledge on the interplay of viral and host factors in CHIKV infection as well as potential targets for antivirals.

#### Viperin, Hsp90 Inhibitors, and Interferons

##### Viperin

Viperin (virus inhibitory protein, endoplasmic reticulum-associated, interferon-inducible) is an interferon (IFN)-induced host cell protein that has come into focus because it is responsible for inhibiting viral replication via multiple pathways. It thus represents an interesting target for antiviral drugs (170). Viperin has been reported to inhibit a broad spectrum of DNA and RNA viruses, including members of the herpesvirus, flavivirus, alphavirus, orthomyxovirus, paramyxovirus, rhabdovirus, and retrovirus family (170). CHIKV infection is also curbed via IFN-induction of viperin and compounds leading to the up-regulation of viperin may present a strategy to manage CHIKV infections. Studies could demonstrate that CHIKV infection is controlled via type I IFNs that induce the interferon-stimulated gene (ISG) RSAD2 (radical SAM domain-containing 2) which encodes viperin (171). Teng et al. showed that mice lacking RSAD2/viperin had a higher rate of CHIKV replication and more severe inflammatory symptoms in the joints. A recent study tried to elucidate the role of viperin in shaping the pathogenic CHIKV-specific CD4 T-cell adaptive immune response during late acute disease phase (172). The group used viperin deficient mice in which CD4 T-cell had been depleted. They could demonstrate that increased late acute joint inflammation was exclusively mediated by CD4 T cells and that Th1-IFNγ-producing T cells played a pivotal role in the joint pathology. Further experiments showed that viperin expression contributes to reducing disease severity in both haematopoietic and non-haematopoietic cells (172).

##### Hsp90 Inhibitors

Chaperones help in the folding, assembly and maturation of host- and viral proteins. Almost all viruses depend on the chaperone Hsp90 (heat shock protein 90) especially during replication to ensure their life cycle (173). This causes viruses to be hypersensitive to Hsp90 inhibition and provides a way to curb virus replication. Compounds interfering in Hsp90 function have a potential as broad-spectrum antiviral drugs, especially since experiments with picornaviruses demonstrated that Hsp90 inhibitors are refractory to the development of drug resistance (174). As mentioned before, Hsp90 also plays an important role during CHIKV replication due to its interaction with the nsP3 and nsP4 of CHIKV. The chaperone furthermore stabilizes CHIKV nsP2 and thus promotes virus replication (65). Studies demonstrated that the Hsp90 inhibitor **geldanamycin** (GA) reduce CHIKV replication, particle formation and infection *in vitro* (65, 66). Yet, inhibiting Hsp90 very often results in toxicity, especially for the liver, presumably because Hsp90 is very abundant in liver cells and interacts with multiple proteins at crucial points in the cellular function. A lot of clinical trials with anti-Hsp90 drugs have been abandoned due to the *in vivo* toxicity (175). This also holds true for GA which is hepatotoxic as well as structurally instable, and thus has so far not been approved for clinical usage (176). Research is currently focussing on developing Hsp90 inhibitors with better pharmacological profile, such as **ganetespib**, which is relatively hydrophobic and less toxic (177). Ganetespib is currently under investigation in phase 1-3 clinical trials for the treatment of breast cancer, small cell lung cancer, acute myeloid leukemia, and myelodysplastic syndrome. However, its potential as an antiviral is not known but might be worth investigating once the drug is approved by the FDA.

Lillsunde et al. (178) investigated the antiviral activity of a number of **marine alkaloid-oroidin analogs** that are synthetic compounds and target the Hsp90. Lillsunde tested the compounds in replicon models against HCV and CHIKV. While 4 compounds selectively inhibited the HCV replicon, the compounds exhibited only moderate selectivity and efficacy against the CHIKV replicon in dose-response and cytotoxicity studies.

##### Interferons

Interferons (IFNs) play a vital role in the innate immune response to counter virus infections and thus have been the subjects of multiple studies. IFNs have been tested widely for their potential use as antivirals against a variety of viruses including HIV, Hepatitis C and B, and Influenza A (179). Type I IFNs [alpha/beta interferon (IFN-α/β)] are produced by the host cell upon sensing virus invasion. IFNs upregulate a variety of interferon-stimulated genes (ISGs). The protein products of the ISGs contribute to countering viral infections by suppressing viral spread and supporting the initiation of adaptive immunity [reviewed in (180)]. IFNs Type I are considered a “standard of care” in suppressing chronic HCV and HBV infections, while Type III IFNs have generated encouraging results as a treatment for HCV infection in phase III clinical trials (181). Various studies have confirmed that alphaviruses are also highly sensitive to the antiviral activity of Type-I IFNs (IFN-α/β) (182, 183).

Briolant et al. (27) compared the antiviral efficacy of IFN-α, glycyrrhizin, 6-azauridine, and RBV of inhibiting CHIKV and SFV infection *in vitro*. When combined with RBV, IFN-α2b had a sub-synergistic antiviral effect on both alphaviruses (27). A more recent study by Gallegos et al. (28) confirmed the highly synergistic effect of RBV and IFN α when administered as combination therapy *in vitro*.

*In vivo* studies with IFN-α/β receptor-deficient mice also demonstrated the importance of IFNs against CHIKV infection. The deficient mice lacked adequate IFN-α/β responses to the viral infection and CHIKV caused haemorrhagic fever, shock, and finally resulted in death (184).

Brehin et al. (185) investigated the role of IFN-induced 2′,5′-Oligoadenylate Synthetase (OAS) protein family in innate immunity to CHIKV. OAS proteins are critical components of innate immunity and the group was able to show that the antiviral actions of IFN-α/β in HeLa cells are mediated due to the induction of these proteins. Various ISGs that affect alphavirus replication have been identified, including ISG15, ISG20, P56, ZAP, and Viperin (185).

#### Tetherin

Tetherin [also known as bone marrow stromal antigen 2 (BST-2)] is a host transmembrane protein with antiviral activity that is induced by IFN. Tetherin binds budded viral particles directly to the plasma membrane (PM) and thus restricts the release of enveloped viruses. The virus particles which are thus bound to the PM can then be endocytosed and degraded (186). Two isoforms of tetherin that differ in length are known. They are referred to as L-(long) and S-(short) tetherin and each has distinct biological properties (187). Tetherin showed antiviral activity against alphavirus release and studies demonstrated that tetherin does not affect viral entry or protein expression. L-tetherin is significantly more efficient in inhibiting the SFV release than the short isoform (186).

In response to this antiviral countermeasure, many viruses have evolved tetherin antagonists. Jones (80) postulated that CHIKV nsP1 is such a BST-2/tetherin antagonist. However, Wan et al. (81) could not confirm Jones' findings and suggested that the sole physical tethering of virus particles to the PM is not sufficient to restrict alphaviruses and that the subsequent virus endocytosis is a requirement for efficient inhibition of alphavirus release.

#### Silvestrol

The natural compound silvestrol (a cyclopenta[b]benzofuran flavagline) is an isolate from plants of the genus *Aglaia* and has been the focus of various antiviral studies over the past 5 years. Flavaglines have been the interest of anticancer research for more than two decades because they display antitumor activity (188). Silvestrol is a highly efficient, non-toxic and specific inhibitor of the host RNA helicase eIF4A (eukaryotic initiation factor-4A), which is part of the heterotrimeric translation initiation complex in eukaryotes (189). The host cell needs the RNA helicase eIF4A to unwind structured 5′-untranslated regions (UTRs) of mRNAs to allow translation. Since 5′-capped viral mRNAs often contain structured 5′-UTRs as well, it has been suggested that RNA viruses which have these structures might depend on eIF4A for their translation. Silvestrol proved to be a successful antiviral in multiple *in vitro* studies against a variety of RNA viruses, such as Ebola, Corona-, Picornaviruses and CHIKV (189–191).

Henß et al. (191) demonstrated that by delaying the protein synthesis of CHIKV nsPs and structural proteins, silvestrol also retarded the innate response to CHIKV infection. By curbing the amount of nsPs, silvestrol reduced CHIKV RNA replication. The compound also decreased the host protein shut-off which was induced by CHIKV infection, probably because of the lower total amount of nsP2. In accordance with this, silvestrol seemed not to impair the IFN-induced STAT1 phosphorylation and eIF2 did not become phosphorylated. All these *in vitro* findings suggest that inhibition of the host helicase eIF4A with silvestrol might be a therapeutic strategy to treat CHIKV infections. Further research is needed to find out how and if silvestrol can actually be of benefit against CHIKV infection *in vivo*.

#### Protein Kinase C Modulators and Plant Extracts

Plants have always been an important source of active substances and to date about 50% of the licensed drugs are natural products or were inspired by them (192). Natural compounds quite frequently have striking differences compared to chemical molecules, which often result in better pharmacological properties (193). The introduction of today's modern drug discovery process has led to a certain neglect of considering plants as a resource for bioactive compounds. But with the technological improvement in the field of natural product isolation, synthesis and screening, the interest in plants as a source for anti-infective natural compounds has been renewed (194).

After the massive CHIKV outbreak in the Indian Ocean region in 2005–2006, a large-scale quest for novel and selective antiviral compounds was initiated. A project called “Biodiversity and emerging viruses in the Indian Ocean: selection of drug candidates targeting the Chikungunya virus” was financially supported by the Center for Research and Monitoring of Emerging Diseases in the Indian Ocean (CRVOI) and carried out from March 2009 to December 2011 (195). Its goal was to find new selective antiviral compounds derived from plants from the Indian Ocean Region, an area with a vast botanical biodiversity. Soon after the program started, virologists, and natural product chemists discovered that the plant family with the most promising components was the *Euphorbiaceae*.

Especially **polycyclic** and **macrocyclic diterpenoids** as well as molecules derived from them came into focus of antiviral research. Within the family of *Eurphorbia* more than several hundred different macrocyclic diterpenoids of interest have been discovered. These molecules possess various types of carbon skeletons (e.g., jatrophane, lathyrane, myrsinane, ingenane, tigliane, daphnane, etc.). More than 20 skeletal types can only be found in this particular plant family (196). These molecules possess a broad structural diversity due to their different macrocyclic skeletons and the various aliphatic and aromatic ester groups.

Macrocyclic diterpenoids have the ability to modulate protein kinase C (PKC) activity (196). Particularly the **phorbol esters** or **phorboids** have a tendency to bind to phospholipid membrane receptors and activate the PKC (197). PKCs are a multigene family of related serine/threonine kinases that are involved in many signal transduction pathways and cellular responses. PKCs play a role in a multitude of cellular functions such as cell mitogenesis, differentiation and apoptosis, smooth muscle contraction, platelet aggregation, tumor-modulation, and anti-HIV activity (198). PKCs are classified into three sub-families with different isoforms depending on the way of their activation. The classical PKC (cPKC) isoforms (α, β, and γ) require calcium (Ca^2+^) and the membrane-embedded ligand diacylglycerol (DAG) for activation, while the novel PKC (nPKC δ, ε, θ, η) are activated by DAG alone. The atypical PKC (aPKC Mζ- ι/λ) are not dependent on either ligand, but on proteins for activation (199).

All PKCs have an N-terminal regulatory moiety with a C1A domain and a C-terminal catalytic moiety for phosphorylation. Conventional and novel PKC isozymes have a second C1 domain (C1B) to which DAG binds (199). Phorbol esters have a two-order higher affinity to the C1B domain of conventional and novel PKC isoforms than DAG. This leads to the activation of the PKCs.

Recently a study reviewed the anti-CHIKV activity of about 80 naturally occurring macrocyclic diterpenes originating from the *Euphorbiaceae* plant family and about 30 commercially available natural diterpenoids (198) (Table 1). Some of these compounds have also been tested against other alphaviruses, like SFV or SINV. Other studies evaluated the antiviral properties of different plant compounds *in vitro* and found out that the phorbol esters **prostratin (12-deoxyphorbol 13-acetate)** and **12-O-tetradecanoylphorbol 13-acetate (TPA)** are potent inhibitors of CHIKV (11, 200). Allard et al. published on the anti-CHIKV properties of **trigocherrierin A**, an unusual chlorinated daphnane diterpenoid orthoester (DDO) from the plant *Trigonostemon cherrieri* (*Euphorbiaceae*), and analog compounds from the same plant (17, 45). Likewise, Nothias-Scaglia et al. found **Phorbol-12,13-didecanoate** to be the most potent inhibitor of CHIKV replication among 29 commercially available natural diterpenoids (201). Phorbol-12,13-didecanoate is structurally related to TPA. Corlay et al. tested **12-O-decanoylphorbol 13-acetate (DPA)**, a molecule that differs from TPA only by the length of the side chain that is attached at C-12 (10 carbons for DPA vs. 14 carbons for TPA) (34). DPA had anti-CHIKV properties but a small SI of 2.0 reflecting a narrow therapeutic window making this compound a poor choice as a future antiviral drug. A novel DDO called **neoguillauminin A** and four **12-deoxyphorbols** from *Euphorbiaceae* plants were recently found to have significant *in vitro* anti-CHIKV properties, three with an SI above 50 (Table 1) (15).

Yet despite the promising results of resent studies, the question of how said compounds manage to curb CHIKV replication has not been fully answered. Most studies assume that PKCs modulation is the key mechanism, but specifics are still outstanding. At the same time, the manner of how PKCs isoforms regulate intracellular signal transduction pathways and influence biological responses is still under heavy investigation and not completely understood. There are hints indicating that different translocation patters of the PKCs might lead to different intracellular signal transduction and cellular functions (202, 203). The cell type in which the PKCs are activated play a role as to how the response affects the organism. Additionally, the chemical properties (e.g., hydrophobicity) of different phorbol esters seem to play a critical role as well, since they induce different translocation patterns of PKCs in the cell. As conventional PKCs depend on plasma membrane bound Ca^2+^ and DAG as ligands, phorbol esters translocate them primarily to the PM, while the novel PKCs only depend on DAG and translocate to the more abundant and diacylglycerol-rich Golgi membrane (199). Studies showed that the stimulation of PKC δ by different phorbol esters induced distinct patterns of enzyme translocation. This indicates that lipophilicity of phorbol esters may contribute to differential PKC δ localization and thus to potentially different biological activities (203). Nothias-Scaglia et al. demonstrated that the HIV-1 and HIV-2 inhibitory effects of phorbol esters were strongly correlated with those of CHIKV (13). This observation is even more interesting since CHIKV and HIV belong to two different virus genera (alphavirus and lentivirus). Thus, the most probable explanation would be a common PKC-based mechanism of action. Yet a broad and potent PKC modulator with very good anti-HIV activity showed no anti-CHIKV activity, which might indicate that different PKC isoforms are involved in the two different virus life cycles. Abdelnabi et al. (33) tried to shed light on the role of PKCs in the cellular antiviral response to CHIKV infection by studying the mechanism of how **prostratin** works as an antiviral against CHIKV. The group found out that different cell lines express varied levels of diverse PKC isoform. Abdelnabi used four different cell lines [buffalo green monkey kidney (BGM) cells, African green monkey kidney cells (Vero cells), human embryonic lung fibroblasts (HEL), and human skin fibroblast cells] and four different CHIKV strains. Prostratin curbed CHIKV RNA synthesis and the production of infectious virus progeny at a post-entry step during virus replication. The antiviral action of the compound was dose- and cell- dependent. The most potent antiviral effect was observed in human skin fibroblast cells which also showed the highest gene expression levels of the classical PKC isoforms (Table 1). The antiviral activity of prostratin was significantly reduced when PKC inhibitors were present. These results suggest that the activation of mostly classical PKCs is the reason for the antiviral effect of prostratin (33).

### Multiple or Unidentified Targets

Many other molecules have been tested against CHIKV and other alphaviruses in the past 5 years, with a special focus on plant extracts or drugs originally licensed for other diseases. Some seemed promising at first but then, upon closer investigation and with different assay methods, turned out to have a narrow SI or bad chemical properties. For some, the mode of action is still unknown. Here only the most recent or promising will be mentioned if they have been subject to repeated studies. For details on their efficacy (see Table 1).

#### Micafungin

Various researchers successfully tested the antifungal drug micafungin against viruses such as CHIKV, SFV, and SINV *in vitro* (35, 159). Micafungin has been licensed for the treatment of invasive candidiasis in 2005 (204, 205). According to Ho et al., micafungin significantly reduced CHIKV infection, cytopathic effects, and progeny yield (35). The question of how micafungin inhibits viral infection is still not answered. It was observed that the drug proved to be more effective in inhibiting CHIKV progeny yield than in reducing RNA replication (35, 159). The researchers thus deducted that micafungin might have a major influence on the later stages of CHIKV infection. On the other hand, the inhibitory effects of micafungin were stronger in the full-time treatment group than in the post-treatment group. This finding allows the speculation that micafungin might target different intracellular events during virus infection, such as viral replication, intracellular and extracellular transmission, and virus stability. The antifungal action of micafungin comes from the non-reversible inhibition of the β-1,3-D-glucan synthase of fungi, thus blocking the cell wall synthesis (206). Since neither mammalian cells nor viruses contain 1,3-beta-D-glucan polymers, the mechanism of action of micafungin still has to be elucidated. On the other hand, the absence of these polymers in mammal cells indicates a lack of mechanism-based toxicity of the drug that might partially account for the good tolerability in patients.

#### Abamectin, Ivermectin, and Berberine

Varghese et al. (36) conducted HTS of about 3000 compounds for their ability to inhibit CHIKV infection. Some of the substances were already licensed drugs or under investigation in clinical trials. With the help of a *Renilla reniformis* luciferase (Rluc) reporter system in baby hamster kidney (BHK-21) cells, Varghese could evaluate the compounds' impact on viral replication. After a second validation with WT and reporter CHIKV infection essays of 25 initial hits, Varghese identified five compounds with the capacity to curb CHIKV replication (36). Among these secondary hit compounds, **abamectin**, **ivermectin**, and **berberine** performed best with an inhibition activity against CHIKV of over 85%. Toxicity evaluations of these three compounds were done in BHK-21 and human hepatocellular (Huh-7.5) cells (Table 1). All three compounds also exhibited antiviral action against other alphaviruses, including SFV and SINV (39).

Abamectin and ivermectin are macrocyclic lactones which originate from the fungus *Streptomyces avemitilis* and are the most commonly used compounds of the avermectin family. Both drugs are potent endo- and ectoparasitic agents with a broad spectrum of activity. Especially ivermectin has been used as an insecticide for vector control and it seems that apart from its insecticide properties against *Aedes* and *Anopheles* species, it also displays antiviral activity against some arboviruses (207). The fact that ivermectin has both mosquitocidal and antiviral action may come in handy for vector control and limiting virus spread as well as infection at the same time. The drug is currently under investigation in a phase 2 clinical trial as a therapeutic for Dengue haemorrhagic fever (ClinicalTrials.gov identifier: NCT03432442). In flaviviruses (DENV, YFV, TBEV) ivermectin inhibits the NS3 helicase activity and thus curbs viral replication (208). The mode of action of abamectin and ivermectin against CHIKV is not clear, but it is being speculated that the drugs inhibit the RNA synthesis and down-regulate the viral protein expression of the nsP1 and nsP3 (36).

Berberine is a plant-derived isoquinoline alkaloid that is also able to inhibit CHIKV replication in a dose-dependent manner. It is believed to curb RNA synthesis and interfere with the viral protein expression (39). However, berberine has a wide range of bioactivities and it is also possible that the alkaloid interferes with host factors which promote CHIKV replication (209). Berberine reduced the virus-induced activation of cellular mitogen-activated protein kinase signaling, a pathway which is relevant for maintaining the viral life cycle. Inhibiting this kinase cascade with specific drugs resulted in a decreased production of CHIKV progeny virions. Varghese tested berberine *in vivo* in a mouse model where it significantly reduced CHIKV-induced inflammatory disease (210). Berberine is currently under clinical investigation in a variety of trials; however, none of them test its use as an antiviral.

#### Coumarin Conjugates

Coumarins can be found in plants as well as certain microorganisms and animals. The (natural and/or synthetic) coumarins have a wide range of biological activities and they are in focus for the therapy of various conditions. A number of coumarins have been found to display antiviral, anticoagulant, anti-inflammatory, antimutagenic, antitumor, antitubercular, central nervous system stimulant, fungicidal or vasodilator activities (211).

Hwu designed and developed 22 compounds that were made up of uracil, arene, and coumarin derivatives (212). He tried to combine the antiviral properties previously described for uracil derivatives and coumarin compounds. Hwu tested the newly designed compounds against CHIKV *in vitro*. Five molecules displayed significant potency against CHIKV (212). In 2019, the same research group published a study after testing 21 new coumarin derivatives against CHIKV *in vitro*. This time coumarin derivatives had been conjugated with guanosine. Hwu had modified the design of the molecules and after HTS, three of these new conjugates were found to inhibit CHIKV in Vero cells with significant potency but with a better SI than the ones tested before (Table 1) (37). From the structure-activity relationship Hwu deduced that the coumarin moiety was essential and the presence of a –OMe group enhanced the antiviral activity. Still, Hwu did not try to elucidate the work mechanism of the antiviral activity of his compounds.

## Discussion

As CHIKV transmission depends on arthropod vectors in a complex interaction between virus host and the environment, a thorough understanding of these interactions is essential for the development of strategies to curb infections and the geographical spread of vectors. Especially climate change is one factor that may help arboviruses manifest in new areas that were formerly unsuitable for their vectors. International travel might further contribute to importing newly emerging arboviral diseases (like CHIK, Zika, or Dengue Fever virus). With autochthonous infections of CHIKV in France and Italy and established populations of *Aedes albopictus* in southern Germany, it is only a question of time until CHIKV manifests in moderate regions (3).

Thus, antiviral research remains of utmost importance to counter CHIKV infection. The different antiviral modes of action (MoAs), direct (by inhibiting the virus themselves), and indirect (by inhibiting host factors), have different merits, but both need to be considered and possibly combined for synergic effects of different MoAs.

A number of directly inhibiting antivirals against CHIKV that were tested *in vitro* were either discovered via *in silico* approach, high throughput screening of libraries or classical pharmacology. Especially plants have been rediscovered as a source for possible antivirals and yielded promising compounds like prostratin. Other drug candidates have been repurposed and are already licensed for the treatment of different viral diseases, e.g., sofosbuvir, ribavirin, and favipiravir. As these molecules have already been intensely evaluated in patients, trials for them against CHIKV in humans may possibly be fast-tracked. Unfortunately, some failed to maintain their efficacy in *in vivo* experiments (e.g., chloroquine and ribavirin), while others (like favipiravir and sofosbuvir) look more promising in animal experiments but still have to be tested against CHIKV in humans.

Despite multiple efforts in antiviral research, there is no standardized protocol for determining efficacy and toxicity. This makes comparison of the different hits impossible. As demonstrated in Tables 1, 2, efficacy and toxicity values vary considerably depending on the assay method, virus strain, and cell line. Some cell lines are refractory to the toxic effects of the molecules, possibly whitewashing the SI of the potential hit. The same applies for the assay methods, where each has its merits and its flaws. The lack of standardization as well as polypharmacology *in vivo* might be reasons why multiple drugs, although having achieved promising results *in vitro*, failed to be of benefit *in vivo*. Standardized efficacy and toxicity assays would help in calculating the SI which in turn is important for selecting molecules to test *in vivo*. So far, there is no defined cut-off for the SI, but a value of ≥10 is usually considered for animal models (39). A more thorough validation of potential hits in pre-clinical studies (e.g., multiple assay methods of selected hits *in vitro*) might help to avoid disappointment in *in vivo* assays.

Furthermore, as CHIKV infection often go hand in hand with other arboviral infections that are transmitted by the same *Aedes* species (e.g., DENV and ZIKV), a panantiviral which shows efficacy against these other viruses would be ideal. Apart from displaying anti-CHIKV activity, sofosbuvir, suramin, favipiravir, ribavirin, 6-azauridine, and ECGC also display antiviral activity against DENV or ZIKV or both *in vitro* (50, 136, 213–215).

Indirect antivirals targeting host factors yielded some promising results *in vitro*, but *in vivo* tests are still outstanding. This approach bears the risk of disrupting the physiological balance of the host factors which might lead to serious adverse effects.

Although research has brought forth a number of promising compounds, most of them still have to be validated *in vivo* and in clinical trials. The past epidemics caused by CHIKV demonstrated the impact a neglected or (re)emerging disease may have on a naïve population. Agents that have the potential to disable a population for a longer period with possible long-term sequelae, pose a vast threat to health and the economy. With no licensed vaccine and no specific antiviral treatment against CHIKF, research in the area of antiviral therapy is of utmost importance and the effort to find a specific treatment should be continued.

## Author Contributions

Both authors contributed to the article and approved the submitted version.

## Conflict of Interest

The authors declare that the research was conducted in the absence of any commercial or financial relationships that could be construed as a potential conflict of interest.
